# THE RISE OF ROBOT PETS AND DISCOURSES OF TECHNO-COMPANIONSHIP IN LATER LIFE

**DOI:** 10.1093/geroni/igz038.083

**Published:** 2019-11-08

**Authors:** Constance Lafontaine

**Affiliations:** Concordia University, Montreal, Québec, Canada

## Abstract

Robot pets of varying degrees of sophistication are advertised as ideal companions for older adults, with claims that they support their emotional and cognitive needs. A new generation of robot pets (e.g., Lovot) is emerging equipped with internet connections, recognition software, surveillance capacity, and AI platforms to mimic desired aspects of animal-human relationships. This paper draws upon promotional materials gathered at the Consumer Electronics Show 2019 and interviews with robot designers, to probe what the robotization of human-animal relationships tells us about shifting notions of companionship through the life course. The argument is that robot pets are inscribed within discourses that instrumentalize human-animal-technology entanglements and position digital technologies as idealized solutions to perceived age-related problems. Theoretical ideas from animals studies add a creative dimension to aging studies to explore what the societal enthusiasm for robot pets reveals about our understanding of the lived material and relational worlds of older adults.

